# Novel Enzymatic Reagentless Glucose Biosensors Based on Noble Metal Nanostructures

**DOI:** 10.3390/polym18111273

**Published:** 2026-05-22

**Authors:** Natalija German, Anton Popov, Almira Ramanaviciene

**Affiliations:** 1 Department of Immunology and Bioelectrochemistry, State Research Institute Centre for Innovative Medicine, Santariskiu 5, LT-08406 Vilnius, Lithuania; 2 NanoTechnas—Center of Nanotechnology and Materials Science, Institute of Chemistry, Faculty of Chemistry and Geosciences, Vilnius University, LT-03225 Vilnius, Lithuania; anton.popov@chgf.vu.lt (A.P.)

**Keywords:** amperometry and cyclic voltammetry, dendritic gold nanostructures, platinum nanostructures, gold nanoparticles, glucose oxidase, poly(1,10-phenathroline-5,6-dione)

## Abstract

Reagentless glucose biosensors with redox mediator—polymerized 1,10-phenanthroline-5,6-dione (pPD)—were developed and electrochemically investigated. Three types of biosensors based on graphite rod (GR) electrodes modified by (i) 13 nm of gold nanoparticles (AuNPs), (ii) electrochemically synthesized dendritic gold nanostructures (DAuNSs), and (iii) platinum nanostructures (PtNSs) were prepared. All electrodes were modified by glucose oxidase (GOx), and the pPD was polymerized for 2 h. Thus, GR/AuNPs/GOx/pPD, GR/DAuNSs/GOx/pPD, and GR/PtNSs/GOx/pPD electrodes were developed and electrochemically characterized. The electrode without noble metal nanostructures (GR/GOx/pPD) was used as the control. The biosensor based on the GR/DAuNSs/GOx/pPD electrode exhibited the best performance, with the sensitivity of 2.58 μA/(mM cm^2^), the linear range up to 93.7 mM, the limit of detection 0.182 mM, the reproducibility and repeatability of 4.99 and 4.80%, and the storage stability (50% of initial current responses (*t*_1/2_)) for up to 19 days. The achieved high resistance to interfering materials enabled precise glucose detection in real samples, including human serum and beverages. The technological solutions presented in this paper are anticipated to provide opportunities and benefits of developing novel enzymatic reagentless glucose biosensors based on noble metal nanostructures for use in clinical assays and general diagnostics, including blood glucose monitoring in people with diabetes.

## 1. Introduction

Currently, there is a growing global interest in the application of high-tech innovations, such as nanotechnology and biotechnology, in practical analysis [1,2,3,4,5]. Sensors and biosensors, which are sophisticated and high-performance laboratory tools, offer quick, accurate, and convenient ways to detect and measure biological interactions and components [1,2,3,4,5,6,7]. Recently, increasing attention has been focused on the monitoring of biological, clinical, environmental, and industrial systems, as their analysis is becoming ever more critical [1,2,3,4,5,6,7,8]. While highly sensitive, reliable, and precise methods are commonly used for detecting trace substances, they can sometimes be effectively replaced by more affordable electroanalytical techniques [6,8]. Consequently, biosensors have emerged as a powerful tool in analysis, driven by rapidly advancing technology and broad applications in medicine, particularly in clinical diagnostics for detecting various diseases, including dental cavities, cardiovascular disease, obesity, and diabetes [6,7]. Diabetes mellitus, commonly known as diabetes, is the most prevalent endocrine disorder affecting carbohydrate metabolism [9,10,11]. It is a chronic condition caused by the improper regulation of blood glucose levels and is a leading contributor to morbidity and mortality worldwide [9,10,11,12,13]. Hypoglycemia (glucose concentration in the plasma is lower than 70 mg/dL (3.88 mM)) in diabetic persons is linked to morbidity and mortality, and presents a significant challenge to maintaining proper glycemic control [12]. Hyperglycemia, defined as a plasma glucose concentration exceeding 126 mg/dL (7 mM), contributes to diabetes-specific microvascular complications in the retina, renal glomerulus, and peripheral nerves, as well as accelerated atherosclerotic macrovascular disease [13]. One of the key factors in managing diabetes is glycemic assessment, which refers to the concentration of glucose in the blood [4,11]. The most commercially successful biosensors measure glucose in blood samples and are based on enzymes such as glucose oxidase (GOx) or glucose dehydrogenase, accounting for approximately 90% of the global biosensor market [14]. Glucose oxidase consists of two protein subunits, each containing a tightly bound and deeply embedded flavin adenine dinucleotide (FAD) cofactor, which is reduced to FADH_2_ during the enzymatic catalytic process [1,15].

To enhance the kinetically challenging electron transfer between FADH_2_ of GOx and the electrode surface [1], mediators are applied, which efficiently transfer electrons [15]. Characterized by fast electron transfer and high stability, 1,10-phenanthroline-5,6-dione (PD) acts as a proton and electron acceptor in FADH_2_ cofactor regeneration and is used in the design of biosensors and biofuel cells [16,17]. The quinone form of 1,10-phenanthroline-5,6-dione (PD_ox_) is reduced via a proton-coupled two-electron transfer to the hydroquinone-type species (H_2_PD_red_), which is subsequently reoxidized at the electrode surface [17]. The application of a suitable immobilization matrix and redox mediator, polymerized 1,10-phenanthroline-5,6-dione (pPD), in the construction of electrochemical glucose biosensors has been published [18,19]. Glucose oxidase catalyzes the formation of hydrogen peroxide (H_2_O_2_), which initiates the synthesis of pPD [18]. The mechanism of the enzymatic synthesis of poly(1,10-phenanthroline-5,6-dione), in which H_2_O_2_ initiates the process, has not yet been described in detail. It can be predicted that it is a radical-mediated step-growth oxidative polymerization of 1,10-phenanthroline-5,6-dione monomers.

The interest to noble metal nanostructures such as the gold and platinum nanoparticles (AuNPs [20,21,22,23,24] and PtNPs [25,26,27,28,29], respectively), gold and platinum nanostructures (AuNSs [30,31,32,33,34,35,36,37,38] and PtNSs [39,40,41], respectively), gold nanotubes and nanowires [42], gold [43] and platinum [44] nanoclusters grew up last decades. Nanostructures are widespread due to their large surface area, catalytic properties, electroactivity, and conductivity [41,42,43,44,45] in the construction of electrochemical bio- [5,11,35], immuno- [22] and geno-sensors [26], bioimaging/diagnostic systems or optoelectronics [34], nanodiagnostic and nanomedicine, photothermal therapy and radiotherapy [25], and electrolysis [46,47]. The reduction of AuCl_4_^−^ and PtCl_6_^2−^ ions to Au^0^ [32,36,37] and Pt^0^ [32,48] takes place during the formation of dendritic gold nanostructures (DAuNSs), and PtNSs, respectively. In the case of DAuNSs, electrochemical synthesis after reduction to Au atoms results in the formation of new clusters on the deposited gold, giving rise to primary, secondary, and tertiary branches [34,38]. The electrical conductivity, anti-interference capability, and storage stability of biosensors can be enhanced by incorporating noble metal nanostructures into the polymer matrix during electrochemical polymerization [19,28].

Glucose biosensors are rightfully considered the most popular on the global market. Effective blood glucose monitoring is essential for patients with diabetes to ensure appropriate treatment. Many glucose monitoring devices exhibit low sensitivity in clinically relevant ranges, insufficient stability due to enzyme leakage or denaturation, and low selectivity in complex biological matrices. The main concept of this paper was to enhance the analytical performance of a glucose biosensor by applying such nanostructures as AuNPs, DAuNSs, or PtNSs in combination with GOx and pPD. The performance of developed electrochemical biosensors based on GR/AuNPs/GOx/pPD, GR/DAuNSs/GOx/pPD, or GR/PtNSs/GOx/pPD electrodes was evaluated for analytical characteristics and storage stability. The suitability of the GR/DAuNSs/GOx/pPD electrode for glucose monitoring in real samples was successfully demonstrated.

## 2. Materials and Methods

### 2.1. Materials

The hexachloroplatinic (IV) acid hexahydrate (H_2_PtCl_6_·6H_2_O), hydrochloric acid, tannic acid, and carbohydrates including D-(+)-glucose, D(-)-fructose, D(+)-mannose, D(+)-galactose, D(+)-xylose, D(+)-saccharose were purchased from Carl Roth GmbH + Co. (Karlsruhe, Germany); an enzyme—glucose oxidase (type VII, from *Aspergillus niger*, 208 units/mg protein)—from Fluka (Buchs, Switzerland); and trisodium citrate dihydrate and potassium chloride (KCl)—from Penta (Praha, Czech Republic). Graphite rod (GR, diameter of 3 mm), 1,10-phenathroline-5,6-dione and tetrachloroauric acid trihydrate (HAuCl_4_·3 H_2_O), 96% ethanol, and human serum (Type AB) were acquired from Sigma-Aldrich (Saint Louis, MO, USA); L-ascorbic acid (AA)—from Fluka Chemie GmbH (Buchs, Switzerland); uric acid (UA) and 25% glutaraldehyde (GA) solution—from AppliChem GmbH (Darmstadt, Germany); and potassium nitrate (KNO_3_)—from Acros Organics (Morris Plains, NJ, USA). Alfa alumina powder (α-Al_2_O_3_, diameter of 0.3 μm, type N) was obtained from Electron Microscopy Sciences (Hatfield, MA, USA); potassium hydroxide—from Reanal (Budapest, Hungary); and hexaammineruthenium(III) chloride (Ru(NH_3_)_6_Cl_3_)—from Fisher Scientific (Waltham, MA, USA). Sodium acetate trihydrate (Reanal (Budapest, Hungary)) and 0.1 M KCl were mixed to prepare the solution of 0.05 M sodium acetate (SA) buffer (pH 6.0). Red wine ‘’Montmeyrac‘’ was purchased from Les Grands Chais de France (Landiras, France), apple juice ‘’Kubuś’’ from Maspex (Olsztynek, Poland), and Coca-Cola from Coca-Cola HBC (Radzymin, Poland).

### 2.2. Modified GR Electrodes Preparation

**Figure 1:**
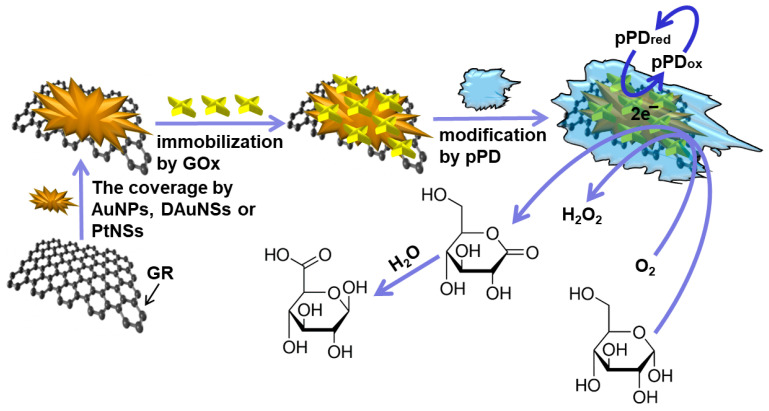
Schematic depiction of the preparation and operation of glucose biosensors based on GR/AuNPs/GOx/pPD, GR/DAuNSs/GOx/pPD, and GR/PtNSs/GOx/pPD electrodes.

To investigate the glucose biosensors presented here, three types of electrodes—(i) GR/AuNPs/GOx/pPD, (ii) GR/DAuNSs/GOx/pPD, and (iii) GR/PtNSs/GOx/pPD—were prepared. The schematic depiction of electrode preparation and operation is illustrated in Figure 1. First, GR (geometric area of 0.071 cm^2^) was polished with fine emery paper and then with an aqueous suspension of α-Al_2_O_3_. GR was washed with distilled water to remove impurities, dried at room temperature (20 ± 2 °C), and placed into a silicone tube.

To prepare the (i) GR/AuNPs/GOx/pPD electrode, the surface of GR was covered by 3 µL of 13 nm AuNPs. The synthesis of the AuNPs solution (pH 6.5) was performed according to the procedure described in the literature [20]. For this purpose, two separate solutions were used, (A) 80 mL of 0.0125% HAuCl_4_ solution and (B) 20 mL of a solution containing 0.2% trisodium citrate dihydrate and 0.00125% tannic acid. Both were heated to +60 °C whilst being stirred continuously with a magnetic stirrer. After that, both solutions were mixed, heated to +98 °C under magnetic stirring, and held at this temperature for a few minutes to yield a red-colored solution. Then, 3 µL of a 25 mg/mL GOx solution was immobilized onto GR modified with AuNPs and allowed to evaporate at room temperature. Covered by AuNPs and GOx, the GR electrode was stored for 15 min in a closed vessel over a 25% GA solution at +20 ± 2 °C to cross-link GOx on the modified GR electrode [20]. Finally, the prepared GR electrode was immersed in the PD polymerization solution for 2 h, consisting of 0.05 M SA buffer (pH 6.0), 6 mM PD dissolved in 96% ethanol, and 0.05 M glucose, at +20 ± 2 °C in the dark.

To fabricate (ii) the GR/DAuNSs/GOx/pPD electrode, the surface of GR was electrochemically coated with DAuNSs from the solution of 6 mM HAuCl_4_ with 0.1 M KNO_3_ using a computerized potentiostat/galvanostat Autolab/PGSTAT 302N (EcoChemie, Utrecht, The Netherlands) with GPES 4.9 software (AUT83239). The electrochemical deposition of DAuNSs was performed at a constant −0.4 V potential vs. Ag/AgCl_(3 M KCl)_ (Metrhom, Herisau, Switzerland) for 400 s by constant potential amperometry (CPA) [30]. For the preparation of (iii) the GR/PtNSs/GOx/pPD electrode aggregates of PtNSs were electrochemically synthesized on the surface of the GR electrode from the solution of 6 mM H_2_PtCl_6_ with 0.1 M KNO_3_ using CPA mode during 100 s and by applying a potential of −0.2 V vs. Ag/AgCl_(3 M KCl)_ [39]. To evaluate the effect of synthesized nanostructures on the current response to glucose, a GR/GOx/pPD electrode was prepared according to the procedures described above, without modification of the electrode surface by nanostructures.

The morphology and size of electrochemically synthesized DAuNSs and PtNSs on the GR were estimated by a high-resolution field emission scanning electron microscope Hitachi SU-70 (FE-SEM, Tokyo, Japan). As seen in Figure S1a,a’, DAuNSs were electrochemically deposited on the surface of GR as elongated, thin, and branched aggregates (the typical observed lateral dimensions for DAuNSs were in the range of 118–411 nm), whereas PtNSs (Figure S1b,b’) formed circular, fused aggregates (the size of PtNSs was from 39 to 500 nm). The further coating of GR modified with DAuNSs or PtNSs, and with GOx and pPD, was performed as described above.

Figure 1 presents the operation of glucose biosensors, where, in the presence of oxygen, the β-D-glucose is oxidized via a two-electron process by GOx to D-glucono-δ-lactone, which is then transformed into D-gluconic acid [15,18]. To ensure the electron transfer between the active site of GOx and the electrode surface, nanostructures [20,33] and redox mediators are used. The stability and anti-interfering capabilities of glucose biosensors could be improved throughout pPD layer formation.

### 2.3. The Evaluation of the Electroactive Surface Area of Modified Electrodes

To calculate the electroactive surface area (EASA) of the fabricated electrodes, measurements were performed by cyclic voltammetry (CV) in a solution of 1 mM Ru(NH_3_)_6_Cl_3_ and 0.1 M KCl, over a potential range from −0.70 to 0.0 V vs. Ag/AgCl_(3 M KCl)_ at scan rates (*υ*) of 0.025, 0.050, 0.075, 0.100, 0.125, 0.150, and 0.175 V/s. The Randles–Sevcik equation [49,50] was used to evaluate the value EASA:

*I*_p_ = 2.69 × 10^5^∙n^3/2^∙EASA∙D^1/2^∙C∙*υ*^1/2^, (1)

where *I*_p_ is the maximum peak current (A), n is the number of electrons transferred in the redox reaction (Ru(NH_3_)_6_^3+^ + e^−^ ⇆ Ru(NH_3_)_6_^2+^ [49]), D is the diffusion coefficient (9.0 × 10^−6^ cm^2^/s [49]), and C is the concentration of electroactive species (0.000001 mol/cm^3^).

### 2.4. The Electrochemical Investigations and the Statistical Evaluation

A three-electrode system, in which a modified GR electrode was applied as the working electrode, a 2 cm^2^ platinum spiral BASi Research Products (West Lafayette, IN, USA) as an auxiliary electrode, and Ag/AgCl_(3 M KCl)_ as a reference electrode, was used for electrochemical investigations performed with a computerized potentiostat/galvanostat Autolab/PGSTAT 302N with GPES 4.9 software. The CV was performed over a potential range of −0.60 to +0.60 V vs. Ag/AgCl_(3 M KCl)_, with a step potential of 2.5 mV and a scan rate of 0.05 V/s in an unstirred 0.05 M solution of SA buffer (pH 6.0). To record current responses of fabricated electrodes in a stirred 0.05 M solution of SA buffer (pH 6.0), the CPA measurements with an applied potential of +0.30 V vs. Ag/AgCl_(3 M KCl)_ were used.

To evaluate the stability of the developed glucose biosensors, modified working electrodes were stored over SA buffer solution (pH 6.0) in a refrigerator (+4 °C) for 59 days. All investigations using CPA were performed at least three times and supplied as the mean value. The intercept, slope, determination coefficient (*R*^2^) of the calibration curve, and the difference between maximum current responses (Δ*I*max) were determined using SigmaPlot 13 (Systat Software Inc., San Jose, CA, USA, demo version). The limit of detection (LOD) was defined as the lowest glucose concentration that yielded a current response greater than the background current response plus 3σ.

### 2.5. Testing of Developed Glucose Biosensor Based on GR/DAuNSs/GOx/pPD in Real Samples

The influence of carbohydrates and electroactive species on a glucose biosensor based on a GR/DAuNSs/GOx/pPD electrode was studied in human serum and beverages using CPA, as previously described in our papers [30,39]. The real samples were stored in the freezer before use. The 10-times diluted in the solution of SA buffer (pH 6.0) human serum, 100-times diluted red wine, and 1000-times diluted apple juice and Coca-Cola were centrifuged for 8 min by the IEC CL31R Multispeed centrifuge from Thermo Industries SAS (Aze Bellitourne, Château-Contier, France) at 14600× *g*. Electrochemical measurements were carried out in human serum after the addition of the 10 mM glucose, 1.0 mM fructose, mannose, galactose, xylose, or saccharose, and finally 10 mM glucose. To evaluate the influence of carbohydrates on glucose biosensors in beverages, electrochemical measurements were performed in red wine, apple juice, and Coca-Cola after the addition of 2.0 mM glucose, 1.0 mM carbohydrates, and finally 6.0 mM glucose. The impact of electroactive species on the glucose biosensor was tested in human serum containing 10 mM glucose, in the presence and absence of 0.01 or 0.1 mM AA and 0.01, 0.05, or 0.1 mM UA. A study was carried out in red wine and apple juice containing 2 mM glucose in the presence and absence of 2 × 10^−4^, 4 × 10^−4^, or 6 × 10^−4^ mM AA. Each investigation with real samples was performed at least three times, and the data were presented as the average of these measurements, with error bars showing the standard deviation. The detection of glucose in diluted real samples was performed to assess the recovery, selectivity, and matrix effects on the developed biosensors.

## 3. Results and Discussions

### 3.1. The Evaluation of EASA of Modified Electrodes

To enhance electron transfer during electrochemical processes and improve the analytical performance of biosensors, noble metal nanostructures are often employed [30,39]. The EASA of developed electrodes was calculated according to the description in Section 2.3. The solution of Ru(NH_3_)_6_Cl_3_ was used as a redox probe, because its oxidation occurs in a diapason where the developed electrodes do not exhibit additional signals. Typical cyclic voltammograms obtained with the developed electrodes are shown in Figure S2. The presented CV results provide indirect, yet well-supported, evidence of electron-transfer behavior. As shown in Figure S2a–d, differently modified electrodes are characterized by rather regular-shaped cyclic voltammograms with clearly defined anodic and cathodic peaks. The cyclic voltammograms registered with GR/AuNPs/GOx/pPD, GR/DAuNSs/GOx/pPD, and GR/PtNSs/GOx/pPD electrodes exhibit higher anodic and cathodic peaks than those registered with the GR/GOx/pPD electrode. The anodic peaks’ currents obtained with GR/GOx/pPD increased from 11.0 to 145 μA, with GR/AuNPs/GOx/pPD from 19.0 to 161 μA, with GR/DAuNSs/GOx/pPD from 20.0 to 180 μA, and with GR/PtNSs/GOx/pPD from 23.0 to 209 μA, respectively, by increasing the scan rate from 0.025 to 0.175 V/s. Higher current peaks may indicate increased EASA or improved conductivity of the modified electrodes. The high anodic currents observed in the presence of AuNPs, DAuNSs, and PtNSs can be attributed to the successful modification of the electrodes with noble metal nanostructures.

**Figure 2:**
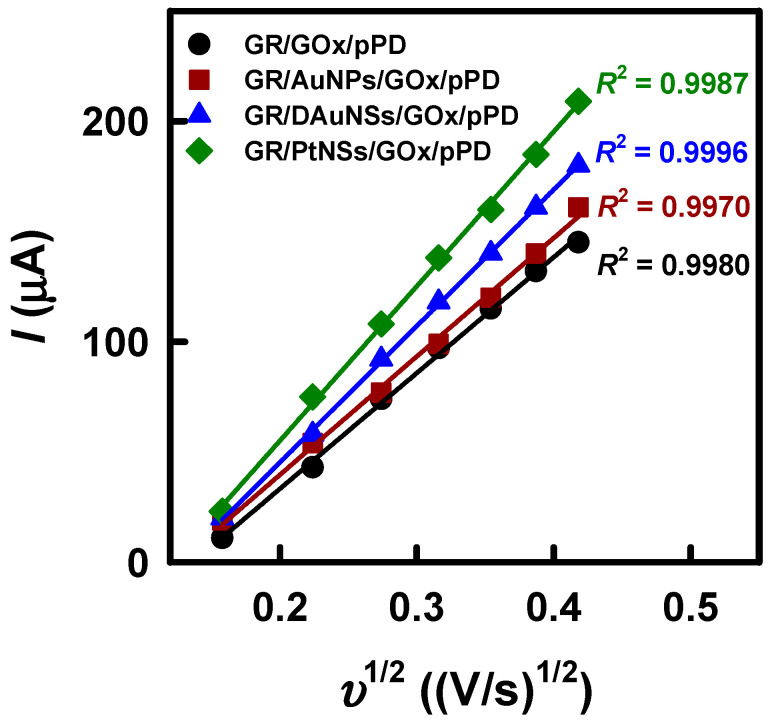
The relationships between the square root of scan rate and the registered peak anodic current for the differently modified electrodes: GR/GOx/pPD (black line), GR/AuNPs/GOx/pPD (brown line), GR/DAuNSs/GOx/pPD (blue line), and GR/PtNSs/GOx/pPD (green line).

The EASA of GR/GOx/pPD, GR/AuNPs/GOx/pPD, GR/DAuNSs/GOx/pPD, and GR/PtNSs/GOx/pPD electrodes was calculated using the Randles–Sevcik Equation (1), where the relationship between the square root of the scan rate and the current response of an anodic peak is the slope of lines (Figure 2).

The Randles–Sevcik equation assumes ideal diffusion conditions. In our case, we used multilayered electrodes of GR/AuNPs/GOx/pPD, GR/DAuNSs/GOx/pPD, GR/PtNSs/GOx/pPD, and GR/GOx/pPD. The limited diffusion through the polymerized mediator layer can affect EASA measurements. The calculated values of EASA for GR/AuNPs/GOx/pPD, GR/DAuNSs/GOx/pPD, GR/PtNSs/GOx/pPD, and GR/GOx/pPD electrodes were considered as an apparent or effective surface area, rather than an absolute physical value. The linear dependence of anodic peak current on the square root of scan rate indicates a diffusion-controlled process. The calculated values of the EASA for GR/GOx/pPD, GR/AuNPs/GOx/pPD, GR/DAuNSs/GOx/pPD, and GR/PtNSs/GOx/pPD electrodes were 0.651, 0.666, 0.767, and 0.869 cm^2^, respectively. The interaction between glucose oxidase and noble metal nanostructures (AuNPs, DAuNSs, or PtNSs) is inferred from the enhanced catalytic response and increased EASA. It shows that noble metal nanostructures are suitable for GOx immobilization due to their morphology and their ability to promote electron transfer between the GOx redox active site and the electrode surface, as reported in other papers [31,39].

### 3.2. The Electrochemical Investigation of Differently Modified Electrodes

**Figure 3:**
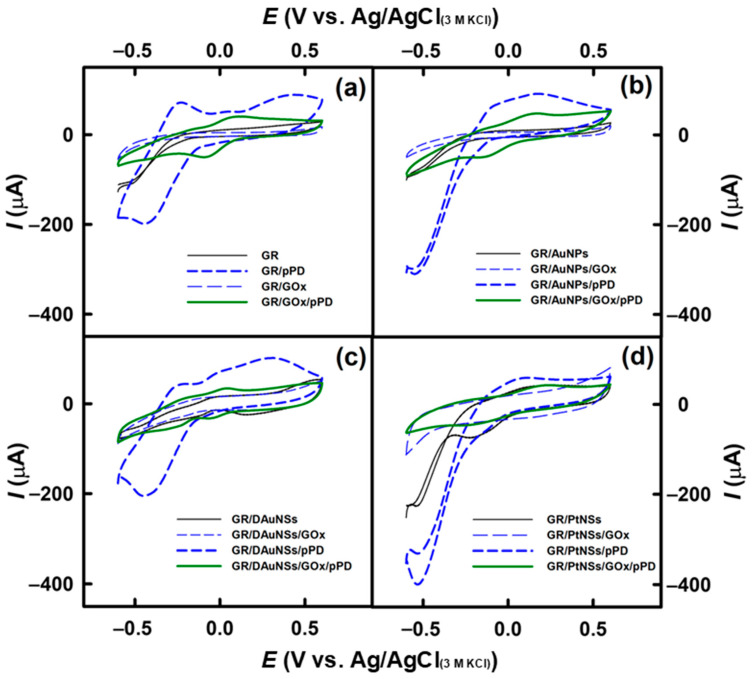
The cyclic voltammograms of glucose biosensors based on unmodified and modified GR/GOx/pPD (**a**), GR/AuNPs/GOx/pPD (**b**), GR/DAuNSs/GOx/pPD (**c**), and GR/PtNSs/GOx/pPD (**d**) electrodes. Cyclic voltammograms were recorded in 0.05 M SA buffer (pH 6.0), at 0.05 V/s of scan rate.

The cyclic voltammogram form and peak shift provide information about the mechanism of the redox reaction and the ability of electron transfer [51]. The electroactivity of differently modified electrodes was investigated by CV according to the description in Section 2.4, and cyclic voltammograms are presented in Figure 3 and Figure S3.

Bare GR, GR/GOx, GR/AuNPs, GR/AuNPs/GOx, GR/DAuNSs, GR/DAuNSs/GOx, GR/PtNSs, and GR/PtNSs/GOx electrodes show narrow, smooth cyclic voltammograms without faradaic response (Figure 3). The formation of the pPD layer on the surface of modified electrodes is confirmed by changes in the cyclic voltammograms, which show oxidation and reduction peaks. The cyclic voltammograms obtained using GR/GOx/pPD, GR/AuNPs/GOx/pPD, GR/DAuNSs/GOx/pPD, and GR/PtNSs/GOx/pPD electrodes showed well-separated anodic and cathodic peaks, indicating the reversibility of the process. The anodic peaks of GR/GOx/pPD, GR/AuNPs/GOx/pPD, GR/DAuNSs/GOx/pPD, and GR/PtNSs/GOx/pPD electrodes were noticeable at +0.115, +0.163, +0.056, and +0.222 V vs. Ag/AgCl_(3 M KCl)_, respectively. The shift in the anodic peak to more positive potentials indicates an increase in charge-transfer resistance due to surface passivation. For further studies, it is advisable to use a potential of +0.30 V vs. Ag/AgCl_(3 M KCl)_ for measurements in CPA mode.

**Figure 4:**
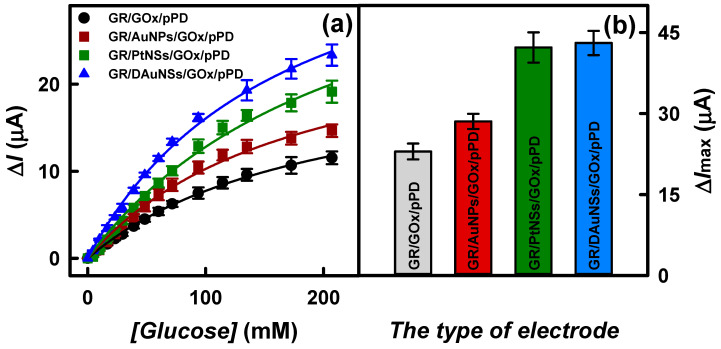
The calibration plots (**a**) and the difference between maximum current responses (**b**) of glucose biosensors based on differently modified electrodes. Measurements were performed using GR/GOx/pPD (black line and gray column), GR/AuNPs/GOx/pPD (brown line and column), GR/PtNSs/GOx/pPD (green line and column), and GR/DAuNSs/GOx/pPD (blue line and column) electrodes at +0.30 V vs. Ag/AgCl_(3 M KCl)_ in 0.05 M SA buffer (pH 6.0).

Nanostructures can affect electron transfer and the sensitivity of biosensors [35,43]. The influence of AuNPs, DAuNSs, and PtNSs on the glucose current responses was investigated using GR/AuNPs/GOx/pPD, GR/DAuNSs/GOx/pPD, and GR/PtNSs/GOx/pPD electrodes in 0.05 M solution of SA buffer (pH 6.0) by CPA, enhancing the concentration of glucose until 207 mM. The GR/GOx/pPD electrode was used as the control. The hyperbolic dependence of current responses on glucose concentration and the diagrams of the difference between maximum current responses for developed biosensors are shown in Figure 4a and Figure 4b, respectively.

As shown in Figure 4, the nanostructures’ modification increases the registered analytical signal compared to the GR/GOx/pPD electrode. The values of Δ*I*max obtained for GR/AuNPs/GOx/pPD (28.5 ± 1.4 μA), GR/PtNSs/GOx/pPD (42.2 ± 2.8 μA), and GR/DAuNSs/GOx/pPD (43.1 ± 2.3 μA) electrodes were 1.24, 1.83, and 1.87 times higher, respectively, than that achieved with the GR/GOx/pPD (23.0 ± 1.5 μA) electrode. This enhancement can be attributed to the increase in the electrode’s electroactive surface area resulting from nanostructure modification.

### 3.3. The Evaluation of Analytical Parameters and Storage Stability of Differently Modified Electrodes

**Figure 5:**
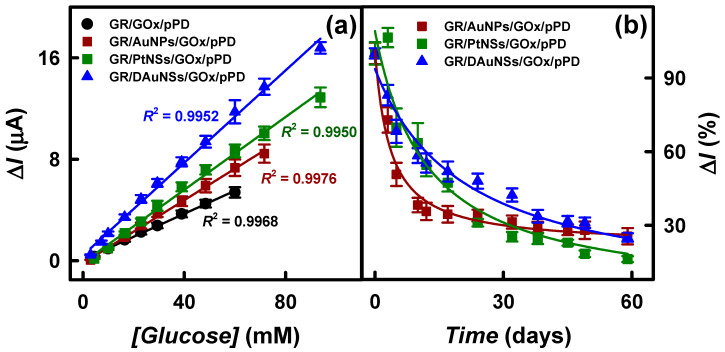
The linear range for GR/GOx/pPD (black line), GR/AuNPs/GOx/pPD (brown line), GR/PtNSs/GOx/pPD (green line), and GR/DAuNSs/GOx/pPD (blue line) electrodes (**a**), and the time variation in current responses (48.4 mM of glucose) for GR/AuNPs/GOx/pPD (brown line), GR/PtNSs/GOx/pPD (green line), and GR/DAuNSs/GOx/pPD (blue line) electrodes (**b**). Current responses were recorded at +0.30 V vs. Ag/AgCl_(3 M KCl)_ in 0.05 M SA buffer (pH 6.0).

To choose the most suitable working electrode for glucose sensing, the analytical performance of the developed electrodes was evaluated. The results are presented in Figure 5a and Table 1.

The linear range (LR) of the developed electrodes was characterized by determination coefficient values of at least 0.9950. It can be argued that incorporating nanostructures extended the linear concentration range of the biosensors. The LR for GR/AuNPs/GOx/pPD (up to 71.6 mM) and GR/DAuNSs/GOx/pPD or GR/PtNSs/GOx/pPD (up to 93.7 mM in both cases) was 1.19 and 1.56 times wider, respectively, than that of GR/GOx/pPD (up to 60.0 mM). The broad LR may be attributed to increased diffusion-related limitation [18].

The obtained LR for GR/DAuNSs/GOx/pPD electrode was 2.40, 4.71, and 374.8 times wider than those previously declared for GR/DAuNSs/(PD/GOx)_3_/Ppy (up to 39.0 mM) [31], for GR/DAuNSs/GOx/Ppy in the presence of PMS as a redox mediator (up to 19.9 mM) [30], and for Au/AuNNs/cysteamine/GOx-BSA-PEGDE (up to 0.25 mM) [52]. The GR/AuNPs/GOx/pPD electrode investigated in this work exhibited LR that was 3.60, 8.95, or 143.2 times wider than those reported for (i) GR/AuNPs_(el. depos.)_/GOx/Ppy in the presence of PMS (up to 19.9 mM) [24]; (ii) GC/OOPpy-AuNPs/GOx (up to 8.0 mM) [21]; and (iii) GC/Au@ILs-polysome/GOx (up to 0.5 mM) [23]. The GR/PtNSs/GOx/pPD electrode developed here was characterized by a 2.40 and 66.9 times wider LR than that obtained for GR/PtNSs/PD/GOx/Ppy (up to 39.0 mM) [39] or for GC/MWCNT/LS/PtNPs_(11.07nm)_/PEI/GOx (up to 1.4 mM) [29].

The glucose biosensors developed in this work, based on GR/AuNPs/GOx/pPD (1.76 μA/(mM cm^2^)), GR/PtNSs/GOx/pPD (2.05 μA/(mM cm^2^)), and GR/DAuNSs/GOx/pPD (2.58 μA/(mM cm^2^)) electrodes, were 1.36, 1.59, and 2.0 times more sensitive, respectively, than the GR/GOx/pPD electrode (1.29 μA/(mM cm^2^)). The LOD for GR/PtNSs/GOx/pPD (0.270 mM), GR/AuNPs/GOx/pPD (0.196 mM), and GR/DAuNSs/GOx/pPD (0.182 mM) electrodes was 3.15, 4.34, and 4.68 times lower, respectively, than that of the GR/GOx/pPD electrode (0.851 mM), demonstrating a clear advantage of biosensor modification by noble metal nanostructures. Furthermore, the LOD for GR/PtNSs/GOx/pPD was 2.08 times lower than that declared for GR/PtNSs/PD/GOx/Ppy (0.561 mM) [39]; the LOD for GR/AuNPs/GOx/pPD was 2.55 times lower than that for GC/OOPpy-AuNPs/GOx (0.5 mM) [21]; and the LOD of GR/DAuNSs/GOx/pPD was 3.75 times lower than that for GR/DAuNSs/(PD/GOx)_3_/Ppy (0.683 mM) [31].

The storage stability of glucose biosensors based on GR/AuNPs/GOx/pPD, GR/DAuNSs/GOx/pPD, and GR/PtNSs/GOx/pPD electrodes was investigated as described in Section 2.4. methodology by keeping electrodes at +4 °C over the SA buffer solution (pH 6.0) for 59 days. The gradual decrease observed for all developed electrodes (Figure 5b) can be attributed to enzyme leakage and polymer degradation [53]. The GR/AuNPs/GOx/pPD, GR/PtNSs/GOx/pPD, and GR/DAuNSs/GOx/pPD electrodes maintained 50% of their initial current responses (*τ*_1/2_) after 6, 15, and 19 days, respectively. The studied glucose biosensor based on GR/DAuNSs/GOx/pPD electrode was 1.27 and 3.17 times more stable than the GR/PtNSs/GOx/pPD and GR/AuNPs/GOx/pPD electrodes, respectively, which can be explained by the better retention of GOx on the DAuNSs and may be a crucial parameter for effective glucose detection in real samples. The GR/DAuNSs/GOx/pPD electrode was characterized by 2.11 times higher storage stability than the GR/DAuNSs/(PD/GOx)_3_/Ppy electrode (τ_1/2_ = 9.0 days) [31]. The presence of DAuNSs helps preserve enzyme activity, and a glucose biosensor based on GR/DAuNSs/GOx retains 50 and 25% of its initial responses after 19 and 59 days, respectively, of storage at +4 °C (Figure 5b). Meanwhile, a glucose biosensor based on GR/PtNSs/GOx/pPD electrode retains 25% of its initial response after 35 days.

**Figure 6:**
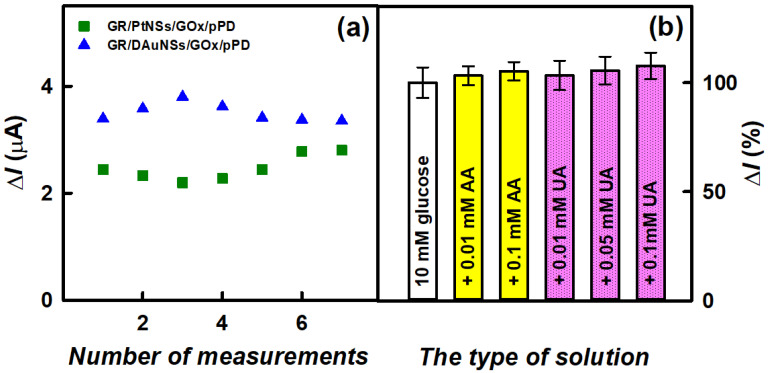
The repeatability study (26.2 mM of glucose) for GR/PtNSs/GOx/pPD (green points) and GR/DAuNSs/GOx/pPD (blue points) electrodes (**a**), and the effect of electroactive species in the human serum sample on the current responses using a glucose biosensor based on GR/DAuNSs/GOx/pPD electrode (**b**). Current responses were recorded in 0.05 M SA buffer (pH 6.0) (**a**) and in 10-times diluted human serum without (white column), and with ascorbic (yellow columns) and uric (pink columns) acids (**b**) at +0.30 V vs. Ag/AgCl_(3 M KCl)_.

The glucose biosensor developed here, based on GR/DAuNSs/GOx/pPD electrode, was characterized by a better value of the reproducibility or batch-to-batch repeatability (4.99% for 26.2 mM of glucose) and the repeatability or intra-assay precision (4.80% for seven measurements) (Figure 6a) than the GR/PtNSs/GOx/pPD electrode, for which the corresponding values were 9.19 and 9.64%, respectively. The repeatability of the GR/DAuNSs/GOx/pPD electrode was better than that of the GR/DAuNSs/(PD/GOx)_3_/Ppy electrode (9.03%) [31]. The time required to reach 95% of the steady-state current response toward glucose for the GR/DAuNSs/GOx/pPD electrode was 5 s, which is 1.6 times faster than that achieved for the previously declared GR/DAuNSs/(PD/GOx)_3_/Ppy electrode (8 s) [31].

Although GR/PtNSs/GOx/pPD exhibited the highest EASA, the superior analytical performance of GR/DAuNSs/GOx/pPD suggests that enzyme and redox mediator immobilization, as well as interfacial electron-transfer kinetics, play crucial roles. The DAuNSs might provide a more favorable matrix for GOx immobilization than PtNSs, owing to their elongated, thin, and branched aggregates. It can facilitate a more uniform distribution and retention of the pPD within the DAuNSs, thereby improving electron transfer between the GOx redox center and the electrode.

### 3.4. The Determination of Glucose in Real Samples

The use of a glucose biosensor combined with DAuNSs and a polymer layer is advantageous due to a broader linear range and improved storage stability, which are key for practical applications. In addition, the polymer layer provides protection against interfering and electroactive substances in real samples [20]. The presence of carbohydrates, as well as hydrophilic antioxidants such as ascorbic acid and uric acid [54,55,56], can interfere with accurate glucose determination because they can be oxidized at positive potentials [6,45,56]. Selectivity toward the analyte is a key parameter that significantly impacts the practical application of biosensors. To evaluate the anti-interfering capability of the glucose biosensor based on GR/DAuNSs/GOx/pPD electrode, the investigations were performed in 10-times diluted human serum, 100-times diluted red wine, and 1000-times diluted apple juice and Coca-Cola, as described in Section 2.5. As shown in Figure S4a–d, the GR/DAuNSs/GOx/pPD electrode is selective for glucose, as no response to 1.0 mM carbohydrates in real samples was observed.

The possible concentration of ascorbic and uric acids in human serum does not exceed 0.141 mM [57] and 0.1 mM [27], respectively, and is much lower than the physiological level of glucose (3–8 mM) [45]. The effect of the presence of the electroactive species in the 10-times diluted human serum sample on the current responses recorded with the GR/DAuNSs/GOx/pPD electrode is shown in Figure 6b. The current response after the addition of 10 mM glucose with 0.01 or 0.1 mM AA increased by 3.0 and 5.0%, respectively, compared to the response without AA. The developed glucose biosensor had 1.2 times greater anti-interference ability against 0.1 mM AA than the GR/pPD/(AuNPs_(13nm)_)pPCA/GOx electrode (6.0%) [19].

The current response after the addition of 10 mM glucose with 0.01, 0.05, or 0.1 mM UA was 3.0, 6.0, or 8.0%, respectively, higher than in the absence of UA. The GR/DAuNSs/GOx/pPD electrode is characterized by higher anti-interfering capability than previously declared GR/DAuNSs/(PD/GOx)_3_/Ppy electrode (the interference of 4.69% for 0.01 mM UA) [31] or GR/DAuNSs/GOx/Ppy electrode in the presence of PMS in solution (the interferences of 4.0 and 16% for 0.01 and 0.1 mM UA, respectively) [30]. The glucose biosensor based on the GR/DAuNSs/GOx/pPD electrode was resistant to AA in diluted beverage samples (Figure S5). After the addition of 2 × 10^−4^, 4 × 10^−4^, or 6 × 10^−4^ mM of AA to samples of red wine and apple juice containing 2.0 mM of glucose, the current responses increased by 5.0, 6.0, 6.0% (Figure S5a) and 4.0, 5.0, 7.0% (Figure S5b), respectively.

The concentration of glucose in the blood of non-diabetic and diabetic persons does not exceed 6 mM [22] and 30 mM [1], respectively. The GR/DAuNSs/GOx/pPD electrode was used to determine glucose in human serum using the ‘standard addition’ method. The total glucose concentration in 10-times diluted human serum was 0.638 mM (the glucose concentration in the used serum was determined by a commercial glucometer (Contour plus, Bayer Consumer Care AG, Basel, Switzerland)). Meanwhile, the detected glucose concentration in 10-times diluted human serum was 0.609 ± 0.013 mM, with a recovery of 95.5% (Figure S6). Results of glucose detection in real samples are presented in Table 2.

The recovery ratios for the glucose biosensor based on the GR/DAuNSs/GOx/pPD electrode in 10-times diluted human serum samples ranged from 95.6 ± 7.0 to 97.3 ± 8.5%. Good ranges of the recovery ratio were obtained in the samples of red wine (from 96.7 ± 4.8 to 98.0 ± 2.2%), apple juice (from 96.0 ± 13.0 to 98.0 ± 5.5%), and Coca-Cola (from 95.0 ± 1.9 to 97.6 ± 10.6%), which demonstrates the suitability for the application of a fabricated biosensor for the diverse applications.

The glucose biosensor developed in this paper is no worse than commercial sensors for glucose detection in human serum. The recovery ratio and relative error of glucose measurements can vary based on the glucose concentration, the method of determination, and the standards set by different countries and agencies. For glucose levels of ≥75 mg/dL (4.17 mM) in the USA, the accuracy criterion according to the International Organization for Standardization 15197:2013 standard is 98 ± 15%; for ≥100 mg/dL (5.55 mM) in Europe—95 ± 15%; and for patients with type 1 diabetes in Australia—99% [58].

The main highlights of developed glucose biosensor based on GR/DAuNSs/GOx/pPD electrode are: (i) good sensitivity (2.58 μA/(mM cm^2^)), reproducibility (4.99%) and repeatability (4.80%); (ii) wide linear range (up to 93.7 mM) and low limit of detection (0.182 mM); (iii) good storage stability (*t*_1/2_ = 19 days) and short duration of measurements (5 s); (iv) high anti-interfering capability and suitability in real analysis; (v) simplicity in construction. While the main focus of this work was the construction of glucose biosensors using various noble metal nanostructures, the practical applications of these biosensors for glucose determination in different samples are also very important. The biosensor fabrication method can be adapted to scalable technologies such as screen printing, enabling cost-effective mass production. In addition, integration with miniaturized microchips and wireless communication modules could enable deployment in portable or wearable devices for real-time glucose monitoring. Further studies are required to validate the scalability of the electrode modification strategy.

## 4. Conclusions

In this paper, we compared the efficiency of relatively inexpensive, simple-to-construct reagentless electrochemical glucose biosensors based on AuNPs, DAuNSs, or PtNSs and enzymatically synthesized pPD. Although all types of developed electrodes offered simplicity of operation and quick response, the GR/DAuNSs/GOx/pPD electrode was characterized by higher sensitivity (2.58 μA/(mM cm^2^)), lower limit of detection (0.182 mM), and better storage stability (*t*_1/2_ = 19 days). Preliminary results on glucose detection in various samples demonstrate the high potential and advantages of the developed nanocomposite-based biosensors, incorporating noble metal nanostructures and pPD, across multiple fields of interest. The proposed technological solutions will contribute to the development of miniature, portable, and real-time biosensors.

## Figures and Tables

**Table 1:** The comparison of analytical performance for glucose biosensors.

Working Electrode; Redox Mediator	LOD (mM)/ Sensitivity (μA/(mM cm^2^))	LR (mM)	Reference
Au/AuNNs/cysteamine/GOx-BSA-PEGDE	0.007/–	0.025–0.25	[52]
GC/MWCNT/LS/PtNPs_(11.07 nm)_/PEI/GOx; –	0.01567/4.77	0.050–1.4	[29]
GC/Au@ILs-polysome/GOx; –	0.02/32.52	0.05–0.5	[23]
GR/DAuNSs/GOx/Ppy; PMS in solution	0.070/59.4	0.1–19.9	[30]
GR/pPD/(AuNPs_(13 nm)_)pPCA/GOx	0.08/0.135 *	0.2–150	[19]
GR/AuNPs_(el. depos.)_/GOx/Ppy; PMS in solution	0.20/21.7	1.0–19.9	[24]
GC/OOPpy-AuNPs/GOx; –	0.5/0.217 *	1.0–8.0	[21]
GR/PtNSs/PD/GOx/Ppy	0.561/5.31	2.00–39.0	[39]
GR/DAuNSs/(PD/GOx)_3_/Ppy	0.683/3.03	2.0–39.0	[31]
GR/DAuNSs/GOx/pPD	0.182/2.58	2.99–93.7	This work
GR/AuNPs/GOx/pPD	0.196/1.76	2.99–71.6	This work
GR/PtNSs/GOx/pPD	0.270/2.05	4.48–93.7	This work
GR/GOx/pPD	0.851/1.29	4.48–60.0	This work

* The sensitivity in μA/mM. Au—gold, Au@ILs-polysome—gold nanoparticles and ionic liquids-based polysome nanocomposites, AuNNs—gold nanopine needles, BSA—bull serum albumin, GC—glassy carbon, LS—lignosulfonate, MWCNT—multi-walled carbon nanotubes, OOPpy—overoxidized polypyrrole, PEGDE—poly(ethylene glycol) diglycidylether, PEI—polyethyleneimine, PMS—phenazine methosulfate, pPCA—poly(pyrrole-2-carboxylic acid), Ppy—polypyrrole.

**Table 2:** Main results obtained with real samples using a biosensor based on the GR/DAuNSs/GOx/pPD electrode (*n*—number of measurements).

The Real Sample	Concentration of Glucose (mM)		Recovery Ratio (%)
	Added	Detected * (*n* = 3)	
Blood serum	9.09	8.69 ± 0.43	95.6
	13.2	12.7 ± 0.31	96.2
	18.6	18.1 ± 1.16	97.3
Wine	2.15	2.08 ± 0.10	96.7
	3.80	3.72 ± 0.05	97.9
	5.50	5.39 ± 0.12	98.0
Apple juice	2.00	1.92 ± 0.26	96.0
	3.40	3.31 ± 0.09	97.4
	5.50	5.39 ± 0.06	98.0
Coca-Cola	2.40	2.28 ± 0.05	95.0
	3.65	3.56 ± 0.10	97.5
	6.15	6.00 ± 0.65	97.6

* Current responses of CPA were recorded at +0.3 V vs. Ag/AgCl_(3 M KCl)_.

## Data Availability

The original contributions presented in this study are included in the article/Supplementary Materials. Further inquiries can be directed to the corresponding authors.

## Supplementary Materials

The following supporting information can be downloaded at: https://www.mdpi.com/article/10.3390/polym18111273/s1, Figure S1: FE-SEM images of electrochemically synthesized DAuNSs (a) and PtNSs (b) on on GR, shown at 20 μm scale for (a,b) and 2 μm scale for (a‘,b‘), respectively. FE-SEM images were acquired at an accelerating voltage of 10 kV and a magnification of 25 k; Figure S2: The cyclic voltammograms of GR/GOx/pPD (a), GR/AuNPs/GOx/pPD (b), GR/DAuNSs/GOx/pPD (c), and GR/PtNSs/GOx/pPD (d) electrodes recorded at scan rates from 0.175 to 0.025 V/s in the solution of 1 mM Ru(NH_3_)_6_Cl_3_ with 0.1 M KCl; Figure S3: The cyclic voltammograms of glucose biosensors based on GR/GOx/pPD (black line), GR/AuNPs/GOx/pPD (brown line), GR/DAuNSs/GOx/pPD (blue line), and GR/PtNSs/GOx/pPD (green line) electrodes registered in 0.05 M SA buffer (pH 6.0), at 0.05 V/s of scan rate; Figure S4: The influence of carbohydrates on the current responses of glucose biosensor based on GR/DAuNSs/GOx/pPD electrode in the samples of human serum (a), red wine (b), apple juice (c), and Coca-Cola (d). Amperograms were registered (a) in 10-times diluted sample of human serum after the addition of the 10 mM glucose, 1.0 mM carbohydrates, and finally 10 mM glucose; (b–d) in 100-times diluted red wine, 1000-times diluted apple juice, or Coca-Cola after the addition of the 2.0 mM glucose, 1.0 mM carbohydrates, and finally 6.0 mM glucose; at +0.30 V vs. Ag/AgCl_(3 M KCl)_ by CPA; Figure S5: The effect of ascorbic acid in the samples of red wine (a) and apple juice (b) on the current responses using a glucose biosensor based on GR/DAuNSs/GOx/pPD electrode. Current responses were recorded in 100-times diluted red wine (a) and 1000-times diluted apple juice (b) at +0.30 V vs. Ag/AgCl_(3 M KCl)_; Figure S6: The determination of glucose in human serum. Measurements were performed on GR/DAuNSs/GOx/pPD electrode at +0.30 V vs. Ag/AgCl_(3M KCl)_ in a 10-times diluted sample of human serum with 0.638 mM of glucose using the ‘standard addition’ method.

## Abbreviations

The following abbreviations are used in this manuscript:

AA	L-ascorbic acid
Ag/AgCl_(3 M KCl)_	Reference electrode
α-Al_2_O_3_	Alfa alumina powder
Au	Gold
AuNPs	Gold nanoparticles
AuNSs	Gold nanostructures
Au@ILs-polysome	Gold nanoparticles and ionic liquids-based polysome nanocomposites
AuNNs	Gold nanopine needles
BSA	Bull serum albumin
C	Concentration of electroactive species
CPA	Constant potential amperometry
CV	Cyclic voltammetry
D	Diffusion coefficient
DAuNSs	Dendritic gold nanostructures
EASA	Electroactive surface area
FAD	Flavin adenine dinucleotide
FADH_2_	Reduced form of flavin adenine dinucleotide
FE-SEM	Field emission scanning electron microscope
GA	Glutaraldehyde
GC	Glassy carbon
GOx	Glucose oxidase
GR	Graphite rod
H_2_O_2_	Hydrogen peroxide
HAuCl_4_·3 H_2_O	Tetrachloroauric acid trihydrate
H_2_PD_red_	Hydroquinone
H_2_PtCl_6_·6H_2_O	Hexachloroplatinic (IV) acid hexahydrate
* I *_ p _	Maximum peak current
Δ*I*max	Difference between maximum current responses
KCl	Potassium chloride
KNO_3_	Potassium nitrate
LOD	Limit of detection
LR	Linear range
LS	Lignosulfonate
MWCNT	Multi-walled carbon nanotubes
n	Number of electrons
* n *	Number of measurements
OOPpy	Overoxidized polypyrrole
PD	1,10-Phenanthroline-5,6-dione
PD_ox_	Quinone
PEGDE	Poly(ethylene glycol) diglycidylether
PEI	Polyethyleneimine
PMS	Phenazine methosulfate
pPCA	Poly(pyrrole-2-carboxylic acid)
pPD	Polymerized 1,10-phenanthroline-5,6-dione
Ppy	Popypyrrole
PtNPs	Platinum nanoparticles
PtNSs	Platinum nanostructures
* R *^2^	Determination coefficient
Ru(NH_3_)_6_Cl_3_	Hexaammineruthenium(III) chloride
SA	Sodium acetate
* τ *_1/2_	50% of initial current responses
UA	Uric acid
* υ *	Scan rate
