# Supplementary material for: Novel Enzymatic Reagentless Glucose Biosensors Based on Noble Metal Nanostructures
Source: Polymers (Basel). 2026 May 22;18(11):1273. doi: 10.3390/polym18111273 (PMC13259085; doi:10.3390/polym18111273)
Supplement: Supplementary file 1 [file polymers-18-01273-s001.zip › polymers-4276951-supplementary.pdf]

Novel Enzymatic Reagentless Glucose Biosensors Based on Noble Metal Nanostructures

Natalija German [1,*], Anton Popov [1,2], Almira Ramanaviciene [1,2,*]

[1] Department of Immunology and Bioelectrochemistry, State Research Institute Centre for Innovative Medicine, Santariskiu 5, LT-08406, Vilnius, Lithuania
[2] NanoTechnas — Center of Nanotechnology and Materials Science, Institute of Chemistry, Faculty of Chemistry and Geosciences, Vilnius University, LT-03225, Vilnius, Lithuania; anton.popov@chgf.vu.lt (A.P.)
* Correspondence: natalija.german@imcentras.lt (N.G.), almira.ramanaviciene@chf.vu.lt (A.R.)

Supplementary Materials

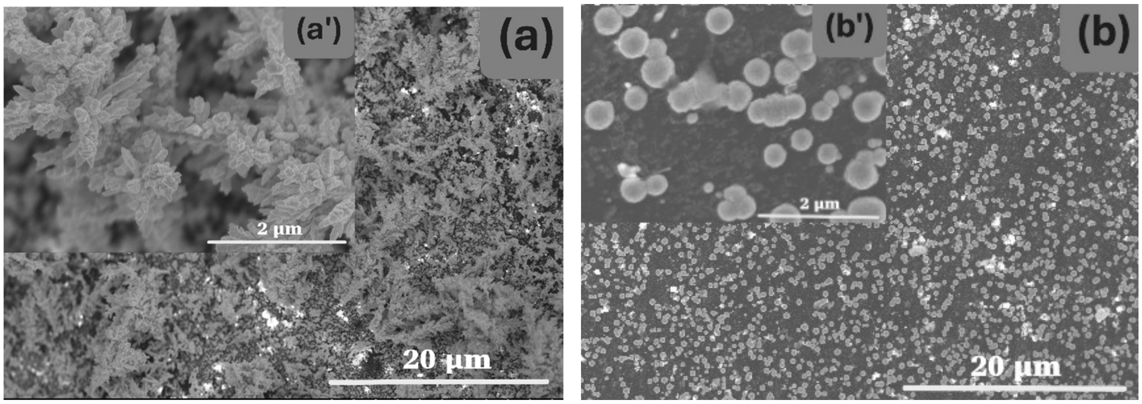

Figure S1. FE-SEM images of electrochemically synthesized DAuNSs (**a**) and PtNSs (**b**) on GR, shown at 20 μm scale for (**a**,**b**) and 2 μm scale for (**a'**,**b'**), respectively. FE-SEM images were acquired at an accelerating voltage of 10 kV and a magnification of 25 k.

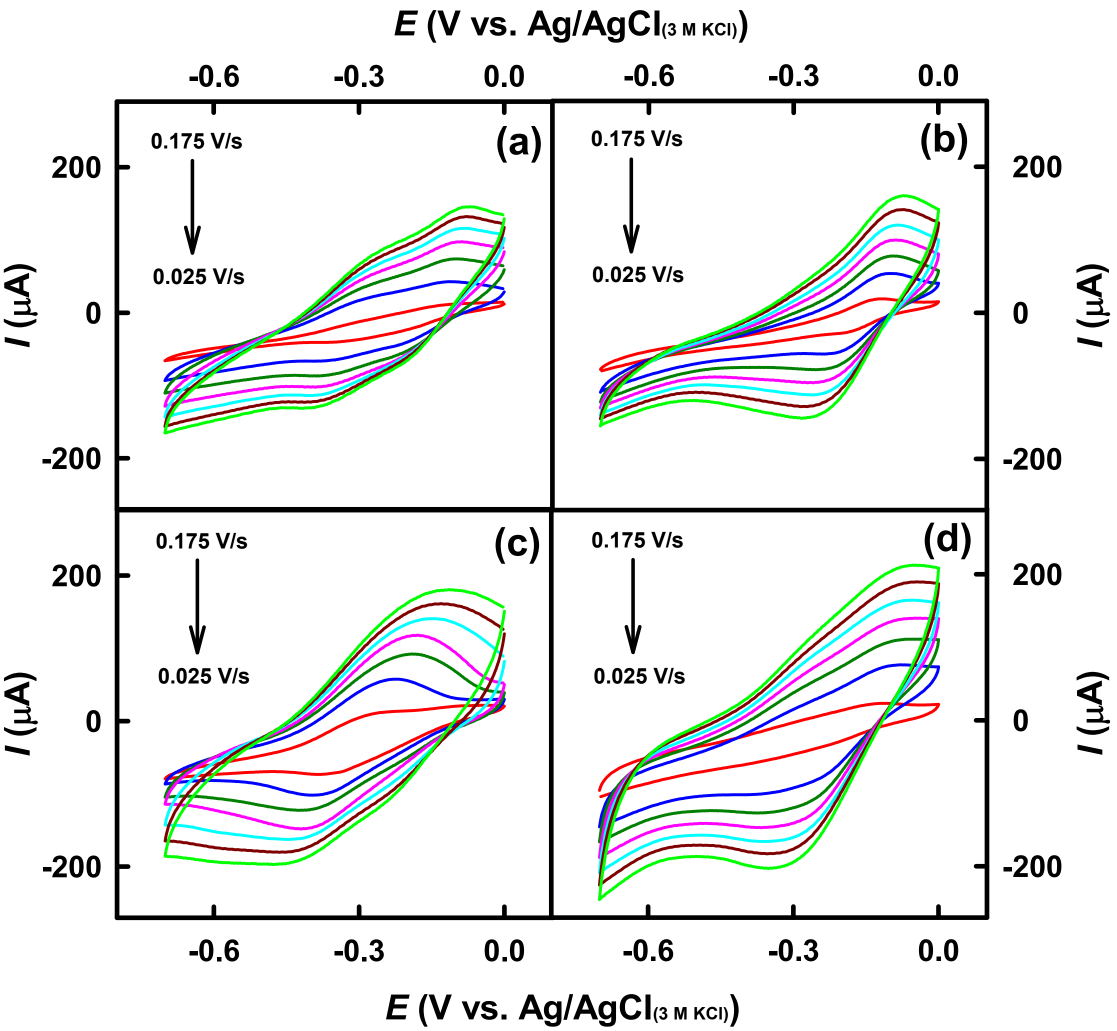

Figure S2. The cyclic voltammograms of GR/GOx/pPD (**a**), GR/AuNPs/GOx/pPD (**b**), GR/DAuNSs/GOx/pPD (**c**), and GR/PtNSs/GOx/pPD (**d**) electrodes recorded at scan rates from 0.175 to 0.025 V/s in the solution of 1 mM $Ru(NH_3)_6Cl_3$ with 0.1 M KCl.

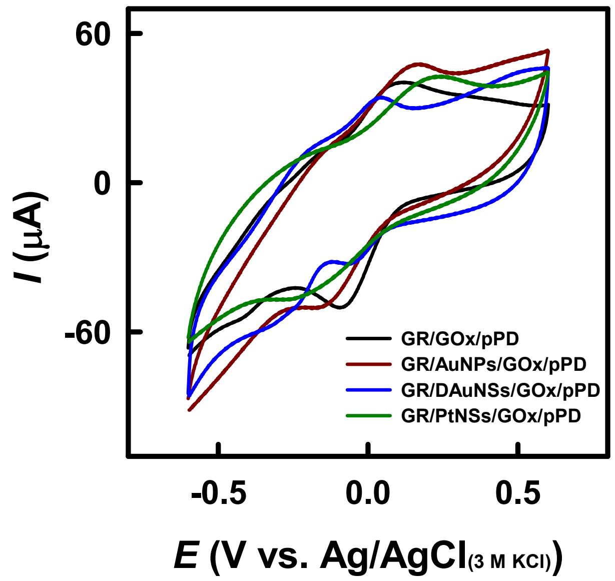

Figure S3. The cyclic voltammograms of glucose biosensors based on GR/GOx/pPD (black line), GR/AuNPs/GOx/pPD (brown line), GR/DAuNSs/GOx/pPD (blue line), and GR/PtNSs/GOx/pPD (green line) electrodes registered in 0.05 M SA buffer (pH 6.0), at 0.05 V/s of scan rate.

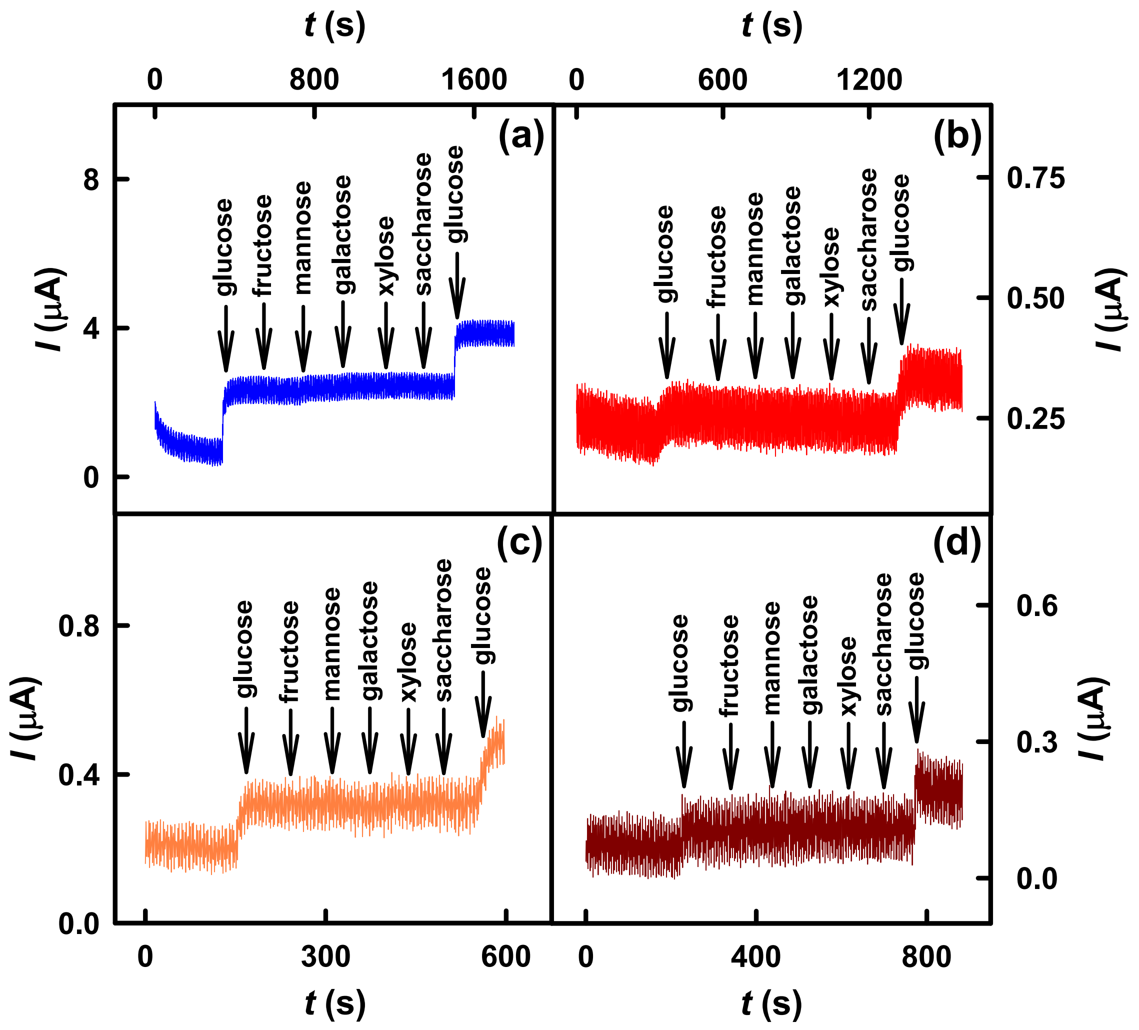

Figure S4. The influence of carbohydrates on the current responses of glucose biosensor based on GR/DAuNSs/GOx/pPD electrode in the samples of human serum (**a**), red wine (**b**), apple juice (**c**), and Coca-Cola (**d**). Amperograms were registered (**a**) in 10-times diluted sample of human serum after the addition of the 10 mM glucose, 1.0 mM carbohydrates, and finally 10 mM glucose; (**b**,**c**,**d**) in 100-times diluted red wine, 1000-times diluted apple juice, or Coca-Cola after the addition of the 2.0 mM glucose, 1.0 mM carbohydrates, and finally 6.0 mM glucose; at +0.30 V vs. $Ag/AgCl_{(3\ M\ KCl)}$ by CPA.

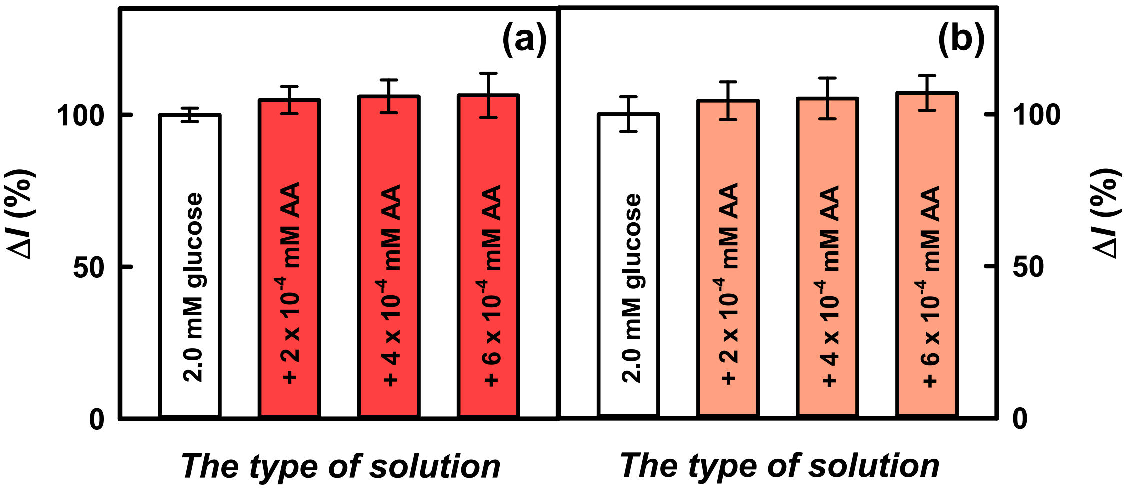

Figure S5. The effect of ascorbic acid in the samples of red wine (**a**) and apple juice (**b**) on the current responses using a glucose biosensor based on GR/DAuNSs/GOx/pPD electrode. Current responses were recorded in 100- times diluted red wine (**a**) and 1000-times diluted apple juice (**b**) at +0.30 V vs. Ag/AgCl$_{(3\ M\ KCl)}$.

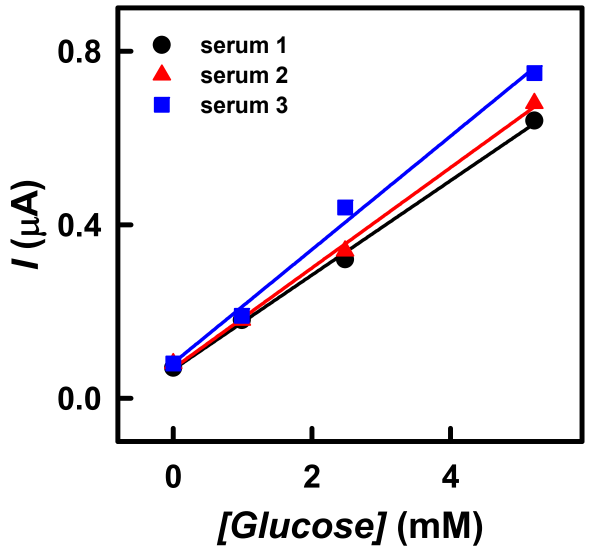

Figure S6. The determination of glucose in human serum. Measurements were performed on GR/DAuNSs/GOx/pPD electrode at +0.30 V vs. Ag/AgCl$_{(3M\ KCl)}$ in a 10-times diluted sample of human serum with 0.638 mM of glucose using the 'standard addition' method.
